# MISSED OPPORTUNITIES IN PREVENTING COGNITIVE DECLINE: UNDERDIAGNOSIS OF POLYCYTHEMIA IN OLDER ADULTS

**DOI:** 10.1093/geroni/igad104.3475

**Published:** 2023-12-21

**Authors:** Blake Eddington, Jeanne Wei, Amanda Pangle, Gohar Azhar

**Affiliations:** University of Arkansas for Medical Sciences, Little Rock, Arkansas, United States; University of Arkansas for Medical Sciences, Little Rock, Arkansas, United States; University of Arkansas for Medical Sciences, Little Rock, Arkansas, United States; University of Arkansas for Medical Sciences, Little Rock, Arkansas, United States

## Abstract

Polycythemia is a hematologic disorder characterized by an abnormally high red blood cell count which can result in serious complications. Microvascular clotting or emboli in polycythemia can reduce brain oxygenation levels with transient ischemic attacks or strokes and lead to subsequent development of vascular dementia. However, the impact of polycythemia on the brain health of older adults may be under-appreciated by primary care providers. The aim of this retrospective study was to investigate the prevalence of missed diagnoses of polycythemia in older adults with cognitive impairment. Electronic medical records (EMR) of patients with cognitive impairment, aged 60-100 years, inclusive of all races, ethnicities, and genders, seen between January 2018-January 2021, were obtained through the University of Arkansas for Medical Sciences database. Cognitive impairment included all subtypes of dementia and delirium with either primary or secondary polycythemia. A total of 9700 patients with cognitive impairment were analyzed for hemoglobin and hematocrit cut-off values for polycythemia. We found 696 instances of comorbid polycythemia of which only 88 (12.6%) had a correct polycythemia diagnosis on record. However, 608 instances (87.4%) had never been identified as having polycythemia either in the problem list or diagnosis. This study highlights the high prevalence of undiagnosed polycythemia in patients with delirium and dementia and underscores the importance of considering polycythemia as a potential etiology, when evaluating cognitive impairment. Early detection and appropriate management of polycythemia in older adults may have implications for reducing the incidence of vascular dementia and improving overall patient outcomes and quality of life.

